# Standard and emerging CMR methods for mitral regurgitation quantification

**DOI:** 10.1016/j.ijcard.2021.01.066

**Published:** 2021-05-15

**Authors:** Benjamin Fidock, Gareth Archer, Natasha Barker, Alaa Elhawaz, Abdallah Al-Mohammad, Alexander Rothman, Rod Hose, Ian R. Hall, Ever Grech, Norman Briffa, Nigel Lewis, Rob J. van der Geest, Jun-Mei Zhang, Liang Zhong, Andrew J. Swift, James M. Wild, Estefania De Gárate, Chiara Bucciarelli-Ducci, Jeroen J. Bax, Sven Plein, Saul Myerson, Pankaj Garg

**Affiliations:** a University of Sheffield, Sheffield, UK; b Sheffield Teaching Hospitals NHS Foundation Trust, Sheffield, UK; c Leiden University Medical Centre, Leiden, the Netherlands; d National Heart Centre Singapore, Singapore; e Bristol Heart Institute, Bristol, UK; f University of Leeds, Leeds, UK; g University of Oxford, Oxford, UK

**Keywords:** Mitral valve insufficiency, Reproducibility of results, Magnetic resonance imaging, 4D, 4 Dimensional, **AoPC**, Aortic Phase-Contrast Forward Volume, **CCC**, Concordance Correlation Coefficient, **CMR**, Cardiac Magnetic Resonance, **FOV**, Field of View, **LVSV**, Left Ventricular Stroke Volume, **MR**, Mitral Regurgitation, **MVR**, Mitral Valve Replacement, **RVSV**, ight Ventricular Stroke Volume, **STJ**, Sino-Tubular Junction, **SV**, Stroke Volume, **VENC**, Velocity Encoding

## Abstract

**Background:**

There are several methods to quantify mitral regurgitation (MR) by cardiovascular magnetic resonance (CMR). The interoperability of these methods and their reproducibility remains undetermined.

**Objective:**

To determine the agreement and reproducibility of different MR quantification methods by CMR across all aetiologies.

**Methods:**

Thirty-five patients with MR were recruited (primary MR = 12, secondary MR = 10 and MVR = 13). Patients underwent CMR, including cines and four-dimensional flow (4D flow). Four methods were evaluated: MR_Standard_ (left ventricular stroke volume - aortic forward flow by phase contrast), MR_LVRV_ (left ventricular stroke volume - right ventricular stroke volume), MR_Jet_ (direct jet quantification by 4D flow) and MR_MVAV_ (mitral forward flow by 4D flow - aortic forward flow by 4D flow). For all cases and MR types, 520 MR volumes were recorded by these 4 methods for intra−/inter-observer tests.

**Results:**

In primary MR, MR_MVAV_ and MR_LVRV_ were comparable to MR_Standard_ (*P* > 0.05). MR_Jet_ resulted in significantly higher MR volumes when compared to MR_Standard_ (*P* < 0.05) In secondary MR and MVR cases, all methods were comparable. In intra-observer tests, MR_MVAV_ demonstrated least bias with best limits of agreement (bias = −0.1 ml, −8 ml to 7.8 ml, *P* = 0.9) and best concordance correlation coefficient (CCC = 0.96, *P* < 0.01). In inter-observer tests, for primary MR and MVR, least bias and highest CCC were observed for MR_MVAV_. For secondary MR, bias was lowest for MR_Jet_ (−0.1 ml, P=NS).

**Conclusion:**

CMR methods of MR quantification demonstrate agreement in secondary MR and MVR. In primary MR, this was not observed. Across all types of MR, MR_MVAV_ quantification demonstrated the highest reproducibility and consistency.

## Highlights

• In primary mitral regurgitation, direct quantification has significant limitations
• MR_MVAV_ method is the most consistent method quantification across all groups.
• All CMR methods are agreeable in secondary and valvular intervention groups

## 1 Introduction

Mitral regurgitation (MR) is the second most common valvular heart disease [1]. The quantification of MR volume categorically differentiates between moderate to severe MR and hence is essential in its assessment; both for conservative management and timing of surgical intervention. Non-invasive imaging by echocardiography or cardiovascular magnetic resonance (CMR) plays an important role in grading MR severity [2].

CMR is established as the reference method for left ventricular volumetric assessment, and more recently, CMR methods of MR volume have shown to predict the timing of intervention and clinical outcomes [[3], [4], [5]]. Overall, CMR provides complimentary clinical assessment when compared to echocardiography [6,7]. There are several ways CMR can quantify mitral regurgitant volume, including emerging techniques which include direct MR jet volume quantification using four-dimensional flow (4D flow) velocity encoded CMR imaging [7,8].

In clinical practice, it is routine to cross-check MR volume quantification between CMR methods to increase confidence in the quantification of MR volume and MR severity. However, the interoperability of these methods remains undetermined. Furthermore, there is limited data on intraobserver and interobserver reliability of different MR quantification methods [9]. A detailed understanding of the intermethod operability and the reliability of each method will help clinical decision making and also will be valuable in designing future prospective clinical studies evaluating outcomes in mitral regurgitation.

Therefore, the main aims of this study were to investigate the agreement of different CMR methods to quantify MR and also assess the reproducibility of each method in patients with primary MR, secondary MR and also in patients who have had mitral valve replacement (MVR).

## 2 Methods

### 2.1 Study population

This is a prospective study which recruited patients from two UK centres with dedicated mitral valve services: Sheffield (*n* = 25) and Leeds (*n* = 10). Patients were identified in outpatient cardiology clinics with an initial diagnosis of MR on echocardiography. Similarly, patients with previous MVR were invited for a CMR study. Sheffield mainly recruited primary MR (*n* = 12) and patients with MVR (*n* = 13). Leeds recruited 10 patients with secondary MR.

The inclusion criteria included: age greater than 18, primary/secondary MR or previous MVR. The exclusion criteria included: patients with valvular stenosis, shunts and contraindication to CMR.

### 2.2 Ethics

This study was approved by the National Research Ethics (17/LO/0283 and 12/YH/0169) in the UK. Written informed consent was obtained from all patients before participation. The study complied with the Declaration of Helsinki.

### 2.3 CMR

At Sheffield, CMR was performed on a 3.0 Tesla Philips Healthcare system (Achieva TX) equipped with a 28-channel coil and Philips dStream digital broadband MR architecture technology. At Leeds, CMR was performed on a similar 1.5 Tesla Philips Healthcare system (Ingenia). Detailed description of CMR acquisition can be found in the online supplementary file.

### 2.4 CMR protocol

The CMR protocol included a baseline survey, cines (vertical long axis, horizontal long axis, short-axis contiguous left ventricle volume stack, 3-chamber and aortic root) and 4D flow. Cines images were acquired during end-expiratory breath-hold with a balanced steady-state free precession, single-slice breath-hold sequence.

### 2.5 4D flow acquisition

For the 4D flow acquisition, VENC setting was set at 150 cm/s. Field-of-view was planned to cover the whole heart, including the aortic root. The 4D flow sequence used echo-planar imaging acceleration factor of 5 with no respiratory gating. This sequence has been validated for valvular flow quantification at both 1.5 T and 3 T field strengths [10,11]. Other scan parameters were: acquired voxel size: 3x3x3mm, reconstructed voxel size: 1.5 × 1.5 × 1.5 mm, echo-time: 3.5 ms, repetition time: 10 ms, flip-angle 10°, the FOV 340 × 340 and 30 cardiac phase. A sense factor of 2 in phase-encoding direction (Anterior-Posterior) was applied. Partial k-space coverage in phase-encoding direction was 90%. Arrhythmias rejection was used in cases where the 4D flow acquisition would halt due to R-R interval variability (occasional case with significant ventricular ectopic or atrial fibrillation). It was then increased to 30% for R-R window variability.

Respiratory motion was monitored continuously by the radiographer performing the examination. Patients were requested to breathe as consistently as possible throughout the three acquisitions. They were given clear instructions not to fall asleep. Average time for 4D flow acquisition was 8 ± 4 min. All 4D flow acquisitions were without contrast.

### 2.6 4D flow corrections

Data pre-processing was done on the scanner for correcting phase offset errors such as eddy currents, Maxwell effects, and encoding errors related to gradient field distortions. Quality checks were performed to ensure that all images met the standards needed for accurate flow quantification. This included checking for slice shift artefacts, velocity aliasing artefacts, and spatial misalignment between the cines and 4D flow dataset. All three-phase directions were screened for aliasing artefact. Particular attention was given to the region of interest: mitral valve area while investigating for any aliasing artefact. If present, this was manually corrected using established phase unwrapping methods [12].

### 2.7 Image analysis

Image analysis was done at Sheffield and Singapore. The analysis was performed offline using the MASS software (version 2019 EXP, LUMC, Netherlands). Ventricular volumes were segmented in the short-axis cine stack as previously described [13,14].

### 2.8 Through-plane aortic flow

For quantification of the aortic forward flow, a fixed reformatted plane was generated in established ways for the quantification of MR. This was performed by generating a reformatted fixed through-plane phase-contrast at the level of sino-tubular junction (STJ) in the ascending aorta. The plane was placed perpendicular to the vessel at the STJ. We used this approach to minimise the overall time of acquisition, and also our previous work demonstrates that two-dimensional phase-contrast acquisition flow is comparable to 4D flow quantified forward flow through the aortic valve [10].

### 2.9 Retrospective valve tracking

On four-chamber cines, the mitral annular plane was identified for the complete cardiac cycle. This plane was checked against the two-chamber, and any angulations were corrected. After ensuring the valve is properly tracked using this plane, we generated a phase-contrast, valvular reformatted plane. On the reformatted valvular plane, we segment the forward flow. Through-plane motion of the valve plane was taken into account for the mitral valve.

### 2.10 MR quantification methods

**Fig. 1:**
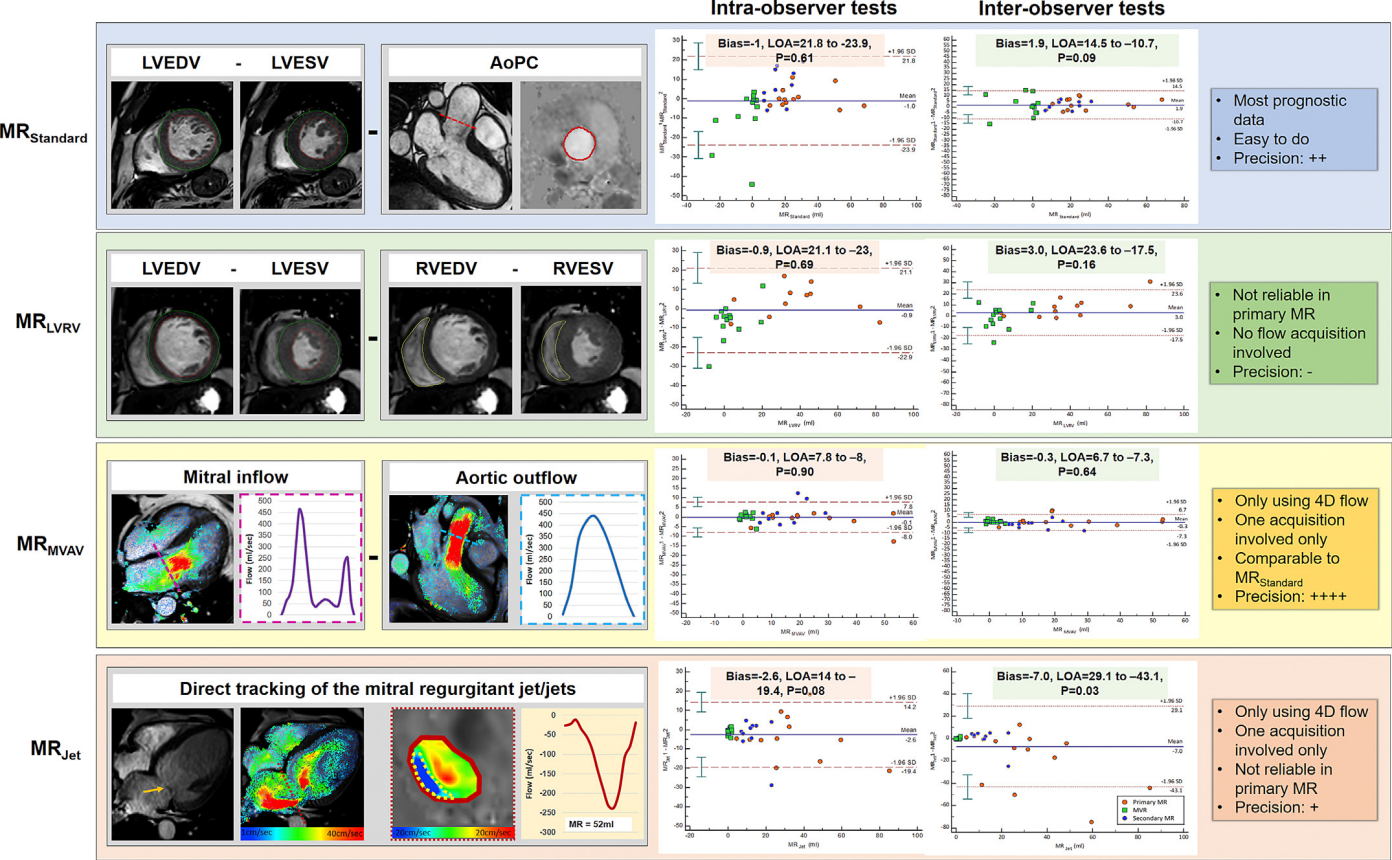
Description of the four CMR MR quantification methods investigated by this study and the intra-observer and intra-observer Bland-Altman analysis results. Characteristics of each quantification method. Orange arrow: prolapse of anterior MV leaflet.

Four CMR MR quantification methods were investigated in this study. Fig. 1 and supplementary table 1 describe the MR quantification methods investigated.

1. **MR**_**Standard**_ (LVSV - AoPC)

This method involved the subtraction of the aortic (systolic) stroke volume obtained by the above described reformatted aortic phase-contrast (AoPC) plane through the sino-tubular junction from the left ventricular stroke volume (LVSV) determined by endocardial segmentation of the short-axis cine stack.

2. **MR**_**LVRV**_ (LVSV - RVSV)

This method involved the subtraction of the right ventricular stroke volume (RVSV) determined by segmentation of the RV in the short-axis cine stack from the LVSV as previously described in **MR**_**Standard**_ method. This method assumes that there is no regurgitation of other valves, and therefore this method was not tested in the secondary MR cohort due to the regular presence of tricuspid regurgitation.

3. **MR**_**MVAV**_ (4D flow mitral forward flow - 4D flow aortic SV)

This method used previously described retrospective valve tracking to quantify mitral and aortic forward flows. Comprehensive retrospective valve tracking procedures are detailed in the online supplementary document.

4. **MR**_**Jet**_ (4D flow direct jet assessment)

MR jets were directly quantified from the 4D flow dataset. Firstly the jet was identified in multiple cine views including two-chamber, three-chamber and four-chamber views. In the four-chamber view, a reformatted plane was placed perpendicular to the regurgitant jet within the left atrium for all phases of the jet life cycle. This was done 1 cm above the mitral valve leaflets for consistency. Dynamic regurgitant jets were quantified by adjusting the reformatted plane throughout the lifecycle of the jet so that the reconstruction remained perpendicular to the direction of flow. If multiple jets existed, each jet was independently segmented and quantified. The total MR volume was calculated as the sum of all MR jets.

### 2.11 Reproducibility tests

Intraobserver tests were performed by two investigators: BF (supervised by AS; >6 years CMR experience) and PG (>6 years CMR experience). Interobserver tests were done by two-second observers NB (supervised by RvdG; >6 years CMR experience) and JMZ (supervised by LZ; >6 years CMR experience). Intra-observer tests were carried out after 1-month of previous analysis. All observers were blinded to the results of other observers.

### 2.12 Statistical analysis

Statistical analysis was performed with IBM SPSS Statistics (version-25). All data were treated as parametric. Continuous variables are expressed as mean ± SD. A repeated-measures ANOVA was performed on demographic comparisons. Intermethod, intra/inter-observer reliability metrics were calculated using concordance correlation coefficient (CCC) analysis and Bland-Altman statistics. CCC measurements of precision (Pearson correlation coefficient) and accuracy (bias correction factor) are detailed in the analysis. The variation between cohorts was quantified with a two-tailed *t*-test. A *p*-value of less than 0.05 was considered statistically significant.

## 3 Results

### 3.1 Demographics

Characteristics of the 35 patients are in Table 1. The average age of patients was 66 ± 11 years. Overall, 66% of patients were male, 50% in the primary MR group, 53.8% in the MVR group and 100% in the secondary MR group. Standard volumetric CMR results are detailed in the online supplementary table 2.

**Table 1:** Study demographics.

	Primary MR *n* = 12	MVR *n* = 13	Secondary MR *n* = 10	P-value
Age	67 ± 11	68 ± 11	62 ± 11	0.97
Gender (% Male)	50%	53.8%	100%	0.03
Height (cm)	167 ± 8	167 ± 9	177 ± 6	0.04
Weight (kg)	75 ± 11	77 ± 11	93 ± 19	0.01
Diabetes Mellitus	1	2	1	0.87
Smoking	7	7	5	0.98
Atrial Fibrillation	3	2	0	0.26
Ischaemic Heart Disease			9	
NYHA Class	2.4 ± 0.9	1.3 ± 0.6	1.7 ± 0.7	0.01

### 3.2 Intermethod agreements

**Fig. 2:**
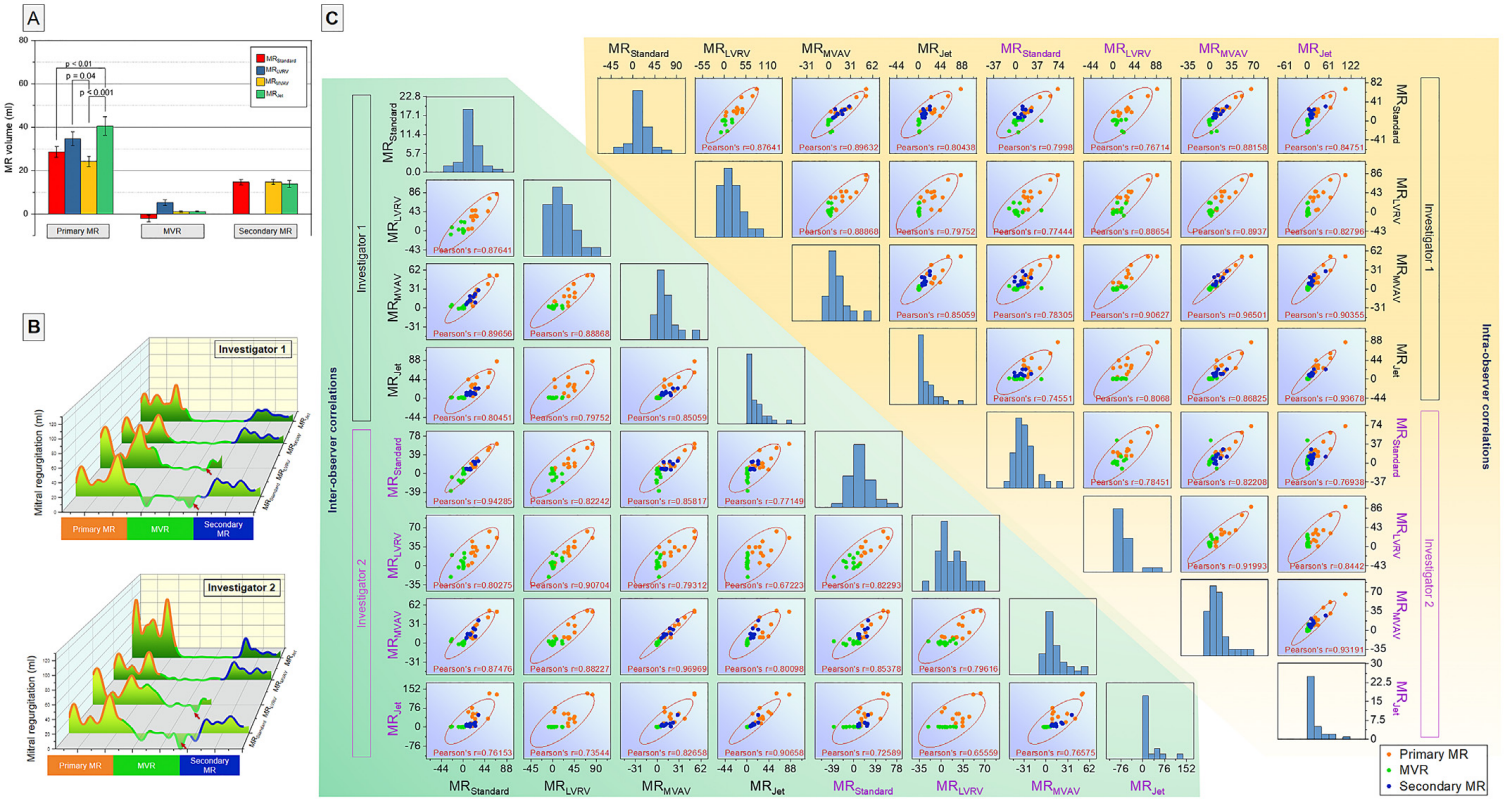
**Panel A.** Inter-method comparison of MR volume quantified for different types of mitral regurgitation. **Panel B.** MR volume quantification data distribution for 2 observers. Note the negative MR volume (red arrows) quantified by the MR_Standard_ and MR_LVRV_. **Panel C.** Scatter matrix of all intra/inter-observer tests. (For interpretation of the references to colour in this figure legend, the reader is referred to the web version of this article.)

For primary MR, the MR_Standard_ and MR_MVAV_ had comparable regurgitant volume (28.6 ± 2.5 ml vs 24.2 ± 2.4 ml, *P* > 0.05). However, MR_LVRV_ was significantly higher than MR_MVAV_. (Fig. 2 Panel A). The MR_Jet_ method regurgitant volume was significantly higher than the MR_Standard_ (*P* < 0.001) and MR_MVAV_ (P < 0.001) with a regurgitant volume of 40.5 ± 4.3 ml.

For the MVR group, the MR_Standard_, MR_LVRV_ and MR_MVAV_ and MR_Jet_ methods all had comparable regurgitant volume (−2.1 ± 1.6 ml vs 5.18 ± 1.3 ml vs 1.0 ± 0.3 ml vs 1.1 ± 0.1 ml respectively). No method was significantly different from any other method. The MR_Standard_ method had a negative regurgitant volume when the aortic stroke volume was greater than the LVSV (Fig. 2 Panel B).

In the secondary MR group, all methods had comparable regurgitant volumes; however, the MR_LVRV_ method was not assessed in secondary MR cases. The MR_Standard,_ MR_MVAV,and_ MR_Jet_ methods had regurgitant volumes of 14.6 ± 1.3 ml, 14.7 ± 1.2 ml and 13.9 ± 1.6 ml, respectively.

Taking into account all the intra/inter-observer tests recording in the study, MR_Standard_ method of regurgitation volume quantification correlated with all the other three methods (MR_LVRV_ *R* = 0.88, *P* < 0.0001; MR_MVAV_ *R* = 0.90, P < 0.0001; MR_Jet_ *R* = 0.80, P < 0.0001). MR_MVAV_ demonstrated the best correlation with MR_Standard_ for both intra−/inter-observer tests (Fig. 2 Panel C).

### 3.3 Intraobserver agreement

Fig. 1 and online supplementary table 3 detail the results of intraobserver agreements. Of all methods tested, the MR_MVAV_ method of regurgitation volume quantification showed the least bias (−0.1 ml, *p* = 0.90), however, no method demonstrated a statistically significant bias (−1.0 ml, −0.9 ml, and − 2.6 ml for MR_Standard_, MR_LVRV_ and MR_Jet_ analysis respectively). In contrast, CCC was poor for MR_Standard_ and MR_LVRV_ quantification methods (0.80 and 0.88 respectively). However, the MR_MVAV_ method demonstrated excellent CCC (0.96) and the MR_Jet_ method demonstrated good CCC (0.91).

### 3.4 Interobserver agreement

Table 2 and Fig. 5 detail the results of interobserver agreements. For primary MR, MR_standard_ and MR_MVAV_ did not demonstrate statistically significant bias (2.9 ml vs 1.0 ml). For both methods, the CCC was substantial (0.95 and 0.96, respectively). The MR_LVRV_ method had a statistically significant bias of 7.5 ml (*p* = 0.02) and a weak CCC of 0.84. The MR_Jet_ method also had a statistically significant bias of −20.1 ml (p = 0.02). This method had a CCC of 0.57 for primary MR. For primary MR, the MR_MVAV_ method had the least bias between observers, whereas the MR_Jet_ method had the most significant bias. The MR_MVAV_ method had minimal bias, and this was less than the MR_Standard_ method.

**Table 2:** Inter-observer reproducibility tests results.

		**Mean Bias**	**Lower Limit**	**Upper Limit**	**P-value**
**Primary MR**	**MR**_**Standard**_	2.9	−7.0	12.8	0.07
	**MR**_**LVRV**_	7.5	−10.7	25.7	0.02
	**MR**_**MVAV**_	1.0	−8.0	10.0	0.46
	**MR**_**Jet**_	−20.1	−71.9	31.7	0.02
**MVR**	**MR**_**Standard**_	1.1	−16.4	18.6	0.66
	**MR**_**LVRV**_	−1.1	−20.9	18.7	0.70
	**MR**_**MVAV**_	0.2	−2.7	3.0	0.72
	**MR**_**Jet**_	−0.2	−1.7	1.4	0.43
**Secondary MR**	**MR**_**Standard**_	1.8	−6.3	9.8	0.21
	**MR**_**LVRV**_				
	**MR**_**MVAV**_	−2.4	−9.4	4.5	0.06
	**MR**_**Jet**_	−0.1	−17.8	17.6	0.97
		**CCC**	**ρ (Precision)**	**Cb (accuracy)**	**P-value**
**Primary MR**	**MR**_**Standard**_	0.95	0.96	0.99	<0.01
	**MR**_**LVRV**_	0.84	0.93	0.90	<0.01
	**MR**_**MVAV**_	0.96	0.96	0.99	<0.01
	**MR**_**Jet**_	0.57	0.82	0.69	<0.01
**MVR**	**MR**_**Standard**_	0.68	0.72	0.95	<0.01
	**MR**_**LVRV**_	0.48	0.51	0.95	0.07
	**MR**_**MVAV**_	0.80	0.85	0.95	<0.01
	**MR**_**Jet**_	0.41	0.43	0.5	0.14
**Secondary MR**	**MR**_**Standard**_	0.85	0.87	0.97	<0.01
	**MR**_**LVRV**_				
	**MR**_**MVAV**_	0.86	0.92	0.94	<0.01
	**MR**_**Jet**_	0.60	0.81	0.74	<0.01

For secondary MR, MR_Standard_, MR_MVAV_ and MR_Jet_ all demonstrated non-significant bias (1.8 ml vs −2.4 ml vs −0.1 ml). MR_LVRV_ was not tested in this group. The CCC for all methods was weak, however, MR_Standard_ and MR_MVAV_ demonstrated similar CCC. For secondary MR, all methods of quantification were comparable, and none showed significant interobserver bias.

For the MVR group, no method demonstrated significant interobserver bias. MR_MVAV_ and MR_Jet_ had a comparable bias of 0.2 ml and − 0.2 ml, respectively. MR_standard_ and MR_LVRV_ also had comparable bias (1.1 ml vs −1.1 ml). In this group, MR_Standard_, MR_LVRV_, MR_MVAV_ and MR_Jet_ all had poor CCC values (0.68 vs 0.48 vs 0.80 vs 0.41). For MVR, all quantification methods had a comparable interobserver bias.

## 4 Discussion

In this study, we comprehensively investigate the intermethod agreement for MR quantification by CMR methods in different types of MR - primary and secondary MR, and in patients with MVR. In addition, this study defines the reproducibility of each CMR method for MR quantification. In primary MR cases, MR_standard_, MR_LVRV_ and MR_MVAV_ demonstrated agreement to each other. However, in MVR and secondary MR, all methods demonstrated reasonable agreement. In both intra/inter-observer tests, MR_MVAV_ demonstrated the least bias and the best concordance correlation suggesting that this is the most precise way to quantify MR.

A previous study by Cawley et al. investigated the reproducibility of MR quantification by CMR in 26 patients with mixed aetiology [15]. They used the MR_Standard_ method to quantify the regurgitant volume and demonstrated similar bias (bias = 0 ml, −18 ml to 17 ml) for intraobserver tests. Śpiewak et al. have previously demonstrated that the exclusion of papillary muscles and trabeculations from the blood pool and the inclusion of them in the LV mass calculation can lead to significantly lower MR volume than when papillary muscles and trabeculations are included in the blood pool [16]. This discrepancy again limits the clinical translation of both MR_Standard_ and MR_LVRV_ for the quantification of MR between two sites using different methods to LV segmentation.

The most established method of primary MR quantification by CMR is MR_Standard_. In a prospective study, Myerson et al. demonstrated that patients with MR_Standard_ ≤ 55 ml had a very high chance of remaining free of symptoms or surgery [3]. Similar findings were noted by Penicka et al. in a study which recruited 258 patients with primary MR [17]. In our study, the method which demonstrates the best association and least difference to MR_Standard_ was MR_MVAV_. Importantly, this method had far better limits of agreement than MR_Standard_. The most plausible explanation for this finding is that this method addresses the issue with the MR_Standard_, which is mainly a higher degree of error due to two acquisitions and multiple slice segmentation for LV stroke volume assessment on short-axis cine stack. MR_MVAV_ method takes advantage of the very accurate transvalvular forward flow assessment using the valve tracking method [18]. In addition, both mitral and aortic flows are quantified for the same averaged cardiac cycles, hence further reducing the chance of errors due to the heart rate variability and spatial miss-alignment. For primary MR, direct quantification with the MR_Jet_ method is susceptible to exaggerated regurgitant volume quantification. We speculate that this is because of variability in defining the plane to quantify the MR jet. Slight variations can result in overestimation as the reconstructed plane can have circulating flow within the LA in direction of the MR jet.

In patients with secondary MR, 4D flow derived MR_Jet_ demonstrated the least bias when compared to other methods. There are several explanations for this observation - firstly, in this study, patients with secondary MR only had mild MR, and hence the regurgitation volume was low, and any relative error using the MR_Standard_ or the MR_MVAV_ methods will appear larger. Secondly, MR_Jet_ method is far less challenging in functional MR cases, as most of them have a central MR jet, which is easy to identify and follow through-out systole.

In patients with MVR, if there is a recurrence of MR, further assessment by imaging is recommended [19]. However, these guidelines acknowledge the fact that MR quantification remains challenging in MVR. All methods described in this study were able to quantify MR in patients with MVR. However, as the regurgitant volume was minimal, we noted inconsistencies with the MR_Standard_ which can again be explained by the inaccuracy of LV stroke volume assessment using standard short-axis cine stack for MR volumes less than 10 ml. Both 4D flow methods appear to be very reliable to quantify MR in this setting.

### 4.1 Clinical perspective

This study demonstrates that the 4D flow CMR derived MR quantification using the MR_MVAV_ method is superior to other CMR methods. 4D flow CMR has advantages over other methods as it is one single acquisition capturing intra-cardiac flow information for the same cardiac cycles. This inherent advantage improves the precision of flow quantification and is not possible by any other non-invasive imaging modality. Moreover, the same 4D flow dataset can be used to quantify MR by two methods - MRJet and MR_MVAV_. With the advent of fast acceleration 4D flow acquisition methods, it now possible to acquire the cross-sectional flow data in 5–10 min. This can be easily adapted in routine CMR protocols after gadolinium injection whilst waiting to do late-enhancement imaging [20]. Quantification of primary MR remains inherently very challenging as the MR jet can be very eccentric. Even though direct MR_Jet_ quantification is possible with 4D flow, it is very time consuming and as this study demonstrates it has poor reproducibility. This is mainly because it requires planning the MR jet plane for every phase of left ventricular systolic acquisition, and then segmenting the mitral regurgitation in the reconstructed plane; both of which, increase the chance of variability between two assessors. Also, in cases where there are multiple dynamic jets, it remains challenging to define the appropriate VENC for MR jet assessment even before acquisition. Hence, MR_MVAV_ method should be the preferred method for improving the precision and reliability for MR quantification. Future studies are needed to evaluate the clinical outcome benefit of using the MR_MVAV_ method for MR quantification.

This study had some limitations. Firstly, direct comparison with echocardiography was not possible as echocardiography did not record MR volume. Secondly, the 4D flow acquisition was without respiratory navigation which may have had an impact on the accuracy of derived velocity/flow parameters. However, studies that carried out a head-to-head comparison of whole-heart 4D flow CMR have demonstrated that for quantification of intra-cardiac flow, both respiratory navigated and non-respiratory navigated 4D flow CMR acquisitions are comparable [21]. Another limitation that could influence the quality of the velocity profile is a low temporal resolution (40 ms).

## 5 Conclusion

CMR methods of MR quantification demonstrate agreement in secondary MR and MVR. In primary MR, this was not observed. Across all types of MR, MR_MVAV_ quantification demonstrated the highest reproducibility and consistency.

## Funding

This work was supported by 10.13039/501100000780European Union funding (H2020 PHC-30–2015, 689617). AR was supported by the 10.13039/100010269Wellcome Trust (206632/Z/17/Z). AS was supported by the 10.13039/100010269Wellcome Trust (205188/Z/16/Z). PG was supported by the 10.13039/501100000691Academy of Medical Sciences (SGL018\1100) and the Wellcome Trust (215799/Z/19/Z, 220703/Z/20/Z).

## Credit author statement

**Gareth Archer** and **Pankaj Garg** recruited and supervised CMR scans. **Norman Briffa**, **Alexander Rothman**, **Rod Hose** and **Pankaj Garg** proposed and designed the study. **Alaa Elhawaz**, **Natasha Barker** and **Benjamin Fidock** supported recruitment, data entry and management. **Pankaj Garg, Benjamin Fidock** and **Gareth Archer** did comprehensive quality checks on all data and handled data management. **Jun-Mei Zhang**, **Liang Zhong**, **Estefania De Garate** and **Chiara Bucciarelli-Ducci** provided external expertise in segmentation and optimization of the study. **Andrew Swift**, **Sven Plein** and **Pankaj Garg** provided clinical reports and assessment. **James M Wild** provided intellectual and infrastructure support. **Rob Van der Geest** provided the software for CMR evaluation. **Rod Hose**, **Norman Briffa**, **Nigel Lewis**, **Ian Hall**, **Abdallah Al-Mohammad, Ever Grech, Jeroen Bax** and **Saul Myerson** provided critical input into the content and discussion regarding the findings of the study. The manuscript, figures and tables were drafted and revised by **Pankaj Garg**. All authors took part in critical review and drafting of the manuscript and have read and approved the final manuscript.

## Declaration of Competing Interest

None.

## Appendix A Supplementary data

Supplementary material Image 1
