# Supplementary material for: Standard and emerging CMR methods for mitral regurgitation quantification
Source: Int J Cardiol. 2021 May 15;331:316–21. doi: 10.1016/j.ijcard.2021.01.066 (PMC8040969; doi:10.1016/j.ijcard.2021.01.066)
Supplement: Supplementary file 1 — Supplementary material [file mmc1.docx]

**Supplementary Data Online**

**Methods**

1. Image acquisition
2. Supplementary Table 1
3. Supplementary Table 2
4. Supplementary Table 3
5. Supplementary Figure 1

**Methods**

**Image acquisition**

**3T Philips System (Sheffield)**

**Cines**

Cines images had a spatial resolution of 2.5 × 2.5 mm2, a pixel size of 1.56× 1.56 mm2, and a slice thickness of 10 mm with contiguous slices for the short axis stack. Other imaging parameters were 30 phases, echo time (TE) = 1.5 ms, repetition time (TR) = 3.05 ms, flip angle= 45°, the field of view (FOV) was 400 mm, and SENSE factor 2–3.

**1.5T Philips System (Leeds)**

**Cines**

Spatial resolution was similar to 3T scans with a slice thickness of 8 mm with contiguous slices for the short axis stack. Other imaging parameters were 30 phases, echo time (TE) 1.5 milliseconds, repetition time (TR) 3 milliseconds, flip angle 60°, the FOV 340mm, and SENSE factor 2.

**Specific adaptations in MVR patients**

In the MVR group, the prosthetic valve resulted in a distortion of the mitral annulus on the four-chamber cines making it challenging to track the mitral valve. In addition, there were pixelation artefacts in the velocity encoded imaging around the region of interest. To circumvent these issues, adaptations were necessary, which are detailed in the online-repository (1) and supplementary Figure 1.

**Supplementary Table 1.** CMR volumetric assessment.

|  | **Primary MR** | **MVR** | **Secondary MR** | **P-value** |
| --- | --- | --- | --- | --- |
| **LVEDV (ml)** | 188.1 ± 44.5 | 134.6 ± 34.6 | 236.1 ± 60.6 | <0.01 |
| **LVESV (ml)** | 84.5 ± 27.3 | 67.7 ± 20.8 | 167.5 ± 59.2 | <0.01 |
| **LVSV (ml)** | 104.1 ± 31.3 | 66.9 ± 20.3 | 68.6 ± 15.2 | <0.01 |
| **LV Mass (g)** | 117.6 ± 25.7 | 104.0 ± 25.3 | 149.4 ±39.2 | <0.01 |
| **LVEF (%)** | 55.3 ± 10.0 | 50.0 ± 7.7 | 30.4 ± 8.9 | <0.01 |
| **RVEDV (ml)** | 162.2 ± 40.4 | 150.8 ± 38.8 | - | 0.48 |
| **RVESV (ml)** | 95.9 ± 38.1 | 87.1 ± 22.5 | - | 0.49 |
| **RVSV (ml)** | 66.3 ± 17.8 | 63.7 ± 19.2 | - | 0.73 |
| **RVEF (%)** | 42.3 ± 11.7 | 42.1 ± 5.3 | - | 0.95 |

LVEDV = left ventricular end diastolic volume; LVESV = left ventricular end systolic volume; LVSV = left ventricular stroke volume; LV mass = left ventricular mass; LVEF = left ventricular ejection fraction; RVEDV = right ventricular end diastolic volume; RVESV = right ventricular end systolic volume; RVSV = right ventricular stroke volume; RVEF = right ventricular ejection fraction.

**Supplementary Table 2.** Overview of all different MR quantification methods in this study.

|  | **Acquisition Time** | **Analysis Time** | **Technical expertise** | **Aliasing issues** | **Precision** |
| --- | --- | --- | --- | --- | --- |
| **MR_Standard_** | **+** | **+** | **+** | **-** | **++** |
| **MR_LVRV_** | **-** | **++** | **++** | **-** | **-** |
| **MR_MVAV_** | **++** | **+++** | **++** | **+** | **++++** |
| **MR_Jet_** | **++** | **++++** | **++++** | **+++** | **+** |

**Supplementary Table 3.** Intra-observer reproducibility test results.

|  | **Mean Bias** | **Lower limit** | **Upper Limit** | **P-value** |
| --- | --- | --- | --- | --- |
| **MR_Standard_** | -1.0 | -23.9 | 21.8 | 0.61 |
| **MR_LVRV_** | -0.9 | --22.9 | 21.1 | 0.70 |
| **MR_MVAV_** | -0.1 | -8.0 | 7.8 | 0.90 |
| **MR_Jet_** | -2.6 | -19.4 | 14.2 | 0.08 |
|  | **CCC** | **ρ (Precision)** | **Cb (accuracy)** | **P-value** |
| **MR_Standard_** | 0.80 | 0.80 | 0.99 | <0.01 |
| **MR_LVRV_** | 0.88 | 0.89 | 0.99 | <0.01 |
| **MR_MVAV_** | 0.96 | 0.97 | 0.99 | <0.01 |
| **MR_Jet_** | 0.91 | 0.93 | 0.98 | <0.01 |

**Supplementary Figure 1.** Adapted methods of mitral forward flow and backward flow quantification in patients with metallic mitral valve replacement. For MR_Jet_ quantification and visualisation during LV systole, a reformatted plane was planned 2cm above the mitral annulus to avoid any artefacts. For mitral forward flow quantification to be used in MR_MVAV_, the reformatted plane was done 1cm below the mitral annulus. All images were checked for any artefacts prior to segmentation.

**
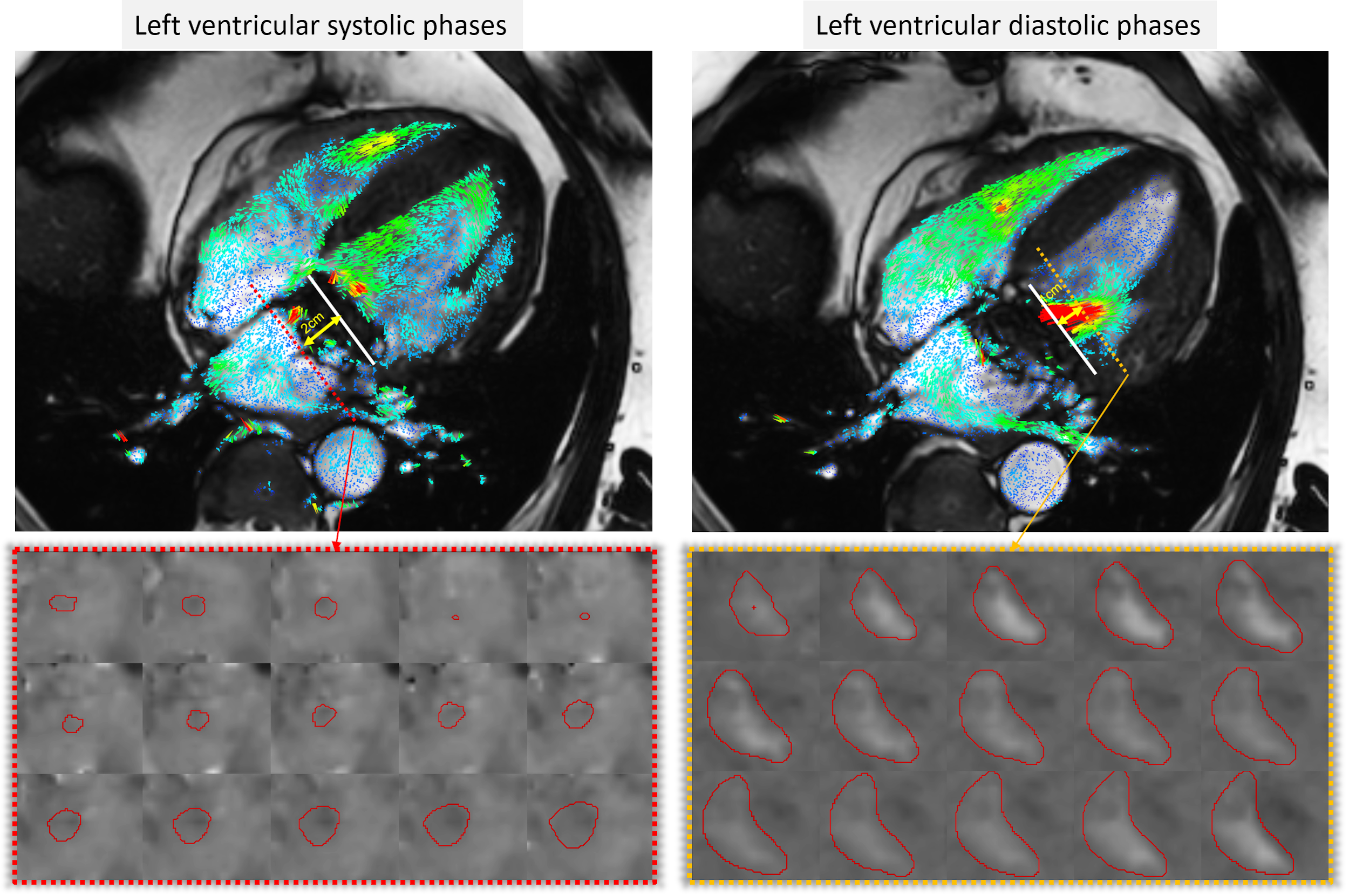
**

References

1. Fidock, B, Garg, P. Method for mitral valve forward and backward flow quantification in patients with prosthetic mitral valve using four-dimensional flow MRI. ResearchGate. 2019;(10.13140/RG.2.2.28833.97123/1).
